# The Book World of Medicine and Science

**Published:** 1897-12-11

**Authors:** 


### The Book World of Medicine and Science.

Health in Africa. By D. Ker Cross, M.B., &c. (London: Jas. Nisbet and Co. 1897. 8vo. Pp. 220. Illustrated. Price 3s. 6d.)

This little volume has been written by Dr. Ker Cross, a medical officer in the service of the British Central Africa Protectorate, and is prefaced by an introduction from the pen of Sir Harry Johnstone, who was till lately, as all the world knows, Imperial Commissioner for British Central Africa. Sir H. H. Johnstone, in commending Dr. Cross's manual to those undertaking pioneer work in trop'cal Africa, insists once again on the enormous natural resources of that fertile region, but is careful to point out how the wealth is only for those who learn to conserve their health. Dr. Cross has written these pages for the lay pioneer rather than for the professional reader, and has included in them an account of most of the ordinary emergencies of medical and surgical practice. The book is simply written, technical phrases are avoided, and elementary facts of physiological and anatomical significance are freely supplied. As might be expected, the best chapters are those dealing with the treatment of purely tropical disorders. Dr. Cross strongly recommends, as do all with special experience, the use of Warburg's tincture in malaria, and advises that drugs should, as much as is possible, be employed in tabloid form. No mention is made of the advantages attending the use of the soluble salts of quinine, or of the value of arsenic in malarial cachexia. Some of the directions for treatment must seem confusing to the unprofessional reader, and the conjunction, in prescriptions, of Latin and English names is not to be commended. Some valuable hints are given on the preservation of health, and the volume is completed by useful lists of drugs, articles of diet, and personal necessities. The technical information is not always correct; elaterium does not, as stated, act powerfully on the liver; and the diagrams are extremely crude. The book as a whole is much disfigured by literary blemishes; "snake virum" and "purient matter" may be misprints, but what can be meant by "sporadic action of muscles" (p. 133)? The famous outburst of Edgar in "King Lear" we all know, but who was the "old heathen" quoted on p. 135, who "long ago observed: Truly the gods make scourges out of our pleasant vices"?

Lawson Tait's Perineal Operations and an Essay on Curettage of the Uterus. By W. J. Stewart McKay, M.B., M.Ch., B.Sc. (London: Baillière, Tindall, and Cox. 1897. Price 3s. 6d.)

This book consists of two parts, quite distinct from one another. The first is a description of Mr. Tait's methods of repairing lacerations of the perineum, the second an essay on curettage of the uterus, the only apparent connecting link between them being the binding of the volume in which they are given to the world. The book, however, is a good one, and the author is to be congratulated on having done what so many medical authors fail to do, *i.e.*, having put his pen down when he had said what he had to say. The details which are given of Tait's operations are so clear that there need be no difficulty in following the various steps described. Unfortunately by some accident in the printing the numbering of the illustrations has gone astray in an extraordinary manner, but by a little care the pictures can be readily identified. The essay on curettage is a very good description of the operation, of its indication, and of its contra-indication and dangers. Among the latter, proper insistance is made on the risks which may arise from curetting the uterus when tubal disease is present. Altogether it is quite worth reading by all who are not familiar with this method of treatment, a method, by-the-by, which in the hands of the unwary is almost as potent for harm as it is for good.

Local Hot Dry Air Treatment in Acute and Chronic Gout. By W. Knowsley Sibley, M.A., M.D., M.R.C.P. (London: The *Lancet* Office, 1897.)

This is a reprint of a paper by Dr. Sibley in the *Lancet*, with the addition of an interesting case of acute, subacute, and chronic gout associated with eczema, treated by the above method. This is a mode of treatment to which we have several times drawn attention (The Hospital, August 1st, 1896, and July 17th, 1897), and is one which is of undoubted efficacy in relieving stiffness in joints and fasciæ arising from various causes. Dr. Sibley, however, shows that it has constitutional effects also, which render it in many cases a valuable therapeutic agency. It must be remembered, however, that the heat without the dryness would not do—in fact, it could not be borne.

The Commercial Uses of Coal Gas. By Thos. Fletcher, F.C.S. (Warrington: Fletcher, Russell, and Co.)

This little book shows the wide field which exists for the use of gas for manufacturing and other purposes, and gives many hints as to the arrangements required. The only points of medical interest are Mr. Fletcher's remarks on the use of flueless stoves, so-called chancel stoves, in churches, and on the employment of gas for sterilisation by heat. In regard to the first matter he shows that the evils which are sometimes charged against them are due to overcrowding and deficient ventilation, and instances the case of a chapel in which less than 100 cubic feet per person were allowed even after including in the calculation all the air space up to the roof 25 feet high, and with a certain humour adds, "The smallest (space) previously recorded appears to be that of a registered common lodging-house of the lowest class, with 240 cubic feet space for each person." In a report recently made on shelters in London several of these institutions were found with less than 170 cubic feet per inmate. But even that is better than the chapel Mr. Fletcher describes. In regard to disinfecting, he says that ovens have been sold for disinfecting clothes in workhouses which were and still are a "constant danger to the community," in that it has been proved that disinfection by dry air is unreliable. The use of gas for this purpose "is out of the question," and every effort should be made to prevent the use of what can only be a dangerous thing. "A disinfector in an infectious diseases hospital, and which does not disinfect, is as dangerous as an unexploded shell in a heap of scrap iron."

